# Clinical Usefulness of Haptoglobin Levels to Evaluate Hemolysis in Recently Transfused Patients

**DOI:** 10.1155/2011/389854

**Published:** 2011-06-26

**Authors:** Shilpi Gupta, Kathleen Ahern, Fadi Nakhl, Frank Forte

**Affiliations:** Department of Hematology and Oncology, Sanford R Nalitt Institute for Cancer and Blood Related Diseases, Staten Island University Hospital, 256 C, Mason Avenue, Staten Island, New York-10305, USA

## Abstract

*Introduction.* Haptoglobin binds the globin portion of free hemoglobin. Serum haptoglobin measurement is used as a laboratory marker for the diagnosis of hemolytic anemia. Since stored blood contains free hemoglobin, transfusion may affect haptoglobin levels. *Study Objectives.* The aim of the study was to evaluate whether serum haptoglobin could be measured to assess hemolysis in recently transfused patients. *Patients and Methods.* Twenty-one patients, receiving more than one unit of packed red blood cells (PRBCs) for presumed nonhemolytic indications, were enrolled. Serum haptoglobin levels were recorded before, immediately after, and 24 and 48 hours after transfusion. *Observations and Results.* Analysis of variance with a repeated measures was used to examine the serum haptoglobin levels at different time periods and no significant difference was found (*P* = .28). *Conclusion.* The results suggest that serum haptoglobin can be used in the diagnosis of hemolysis in patients receiving multiple units of PRBC.

## 1. Introduction

Haptoglobin, an alpha2 globulin, which functions to bind the globin portion of free hemoglobin in the blood, is an acute phase reactant. The half-life of serum haptoglobin is approximately five days [1], but in the presence of free hemoglobin (e.g., intravascular hemolysis), the hemoglobin-haptoglobin complex which forms is rapidly cleared from the serum by the monocyte-macrophage system with resultant serum haptoglobin levels that are low or absent. The hemoglobin-haptoglobin complex also prevents renal tubular injury by inhibiting hemoglobin escape through the glomerulus [2–5]. Thus, an increased burden of free plasma hemoglobin rapidly causes a marked decrease in measured serum haptoglobin (normal levels 36–195 mg/dL).

The measurement of serum haptoglobin is used as one of the laboratory markers for the diagnosis of hemolytic anemia. Studies have revealed the effect of prolonged storage on the amount of free hemoglobin in the stored blood. Nishiyama looked at the effect of blood transfusion on haptoglobin and free hemoglobin. The author noted that as the age of stored blood increased the detection rate of free hemoglobin also went up [6].

In this study we recorded the pre- and posttransfusion haptoglobin levels in patients receiving multiple blood transfusions for presumed nonhemolytic anemia to see whether multiple transfusions lowered haptoglobin values sufficiently to negate its usefulness in diagnosing hemolytic anemia in transfused patients.

## 2. Study Objectives

The aim of the study was to evaluate whether serum haptoglobin could be measured to assess hemolysis in recently transfused patients.

## 3. Methods

### 3.1. Patients

Patients for whom a type and cross-match request for two or more units of PRBC transfusion was made from September 2007 to June 2008 were identified from the blood bank at Staten Island University Hospital. Of these, the patients who were eighteen years of age or older were approached for enrollment in the study. Patients with a history of blood transfusion in the past four months, history of cardiac valve replacement, evidence of hepatomegaly, splenomegaly, and/ or icterus on physical examination, those with abnormal liver function tests, or of any other evidence of hemolysis at the time of enrollment in the study were excluded. Written informed consent for participation in the study and for blood transfusion was obtained. Patients could withdraw from the study at any time. Any patient who developed signs of hemolysis during the study period was removed, and the serum haptoglobin levels were not used in statistical analysis.

### 3.2. Procedure

Once informed consent was obtained and an appropriate indication for blood transfusion was documented, blood samples were obtained from the patients by a peripheral venipuncture, which was performed maintaining aseptic technique. The serum haptoglobin levels were measured four times for each patient: once prior to the first transfusion and three times after the last transfusion (immediately posttransfusion and twenty four and forty eight hours after transfusion). These times were decided based on the half-life of haptoglobin and the hemoglobin- haptoglobin complex. For the pretransfusion measurement of serum haptoglobin, the sample for type and cross-match was used as often as possible to avoid additional venipunctures. The age of each unit of packed red blood cell transfused was also recorded.

### 3.3. PRBC Transfusion

The PRBC transfusion was performed by the nurse caring for the patient as per recommended guidelines. The PRBCs were obtained from the hospital blood bank. The preservative used in the PRBC's was CPD (citrate phosphate dextrose). The units transfused were identified after appropriate type and cross-matching in the blood bank. The study investigators were not involved in selection or transfusion of the PRBC's.

### 3.4. Measurement of Serum Haptoglobin

The serum haptoglobin levels were measured in the hospital laboratory using a Beckmen Coulter Unicel DXC by combining an antihaptoglobin antibody with the haptoglobin molecule, forming an insoluble complex. This antigen-antibody complex was measured by optical absorbance at 340 nanometers using turbidimetry principle [7]. The serum haptoglobin was reported in milligrams per deciliter. The normal range of serum haptoglobin in our hospital laboratory is 36–195 mg/dL.

### 3.5. Statistics

We used analysis of variance with a repeated measure to examine group differences in serum haptoglobin levels over the various time intervals. Univariate paired *t*-test was used to examine differences from pretransfusion to posttransfusion haptoglobin levels. Fisher Exact was used for categorical data. Statistical significance was set at less than or equal to 0.05. Data was examined using Number Cruncher Statistical System (2004). The sample size of twenty-one patients measured four times with the repeated measures Anova was calculated based on a moderate effect size of 0.2.

## 4. Observations and Results

A total of twenty-one patients received forty-seven units of PRBC. The most common indication for transfusion was acute blood loss for gastrointestinal bleeding seen in fifteen patients, of which eleven patients had hemorrhoidal bleeding and four patients had an unidentified gastrointestinal source of bleeding. Three patients had heavy menstrual bleeding from uterine fibroids, one patient was involved in a motor vehicle accident resulting in massive blood loss, and two patients were admitted to the critical care unit with acute respiratory failure from aspiration pneumonia. Although serum haptoglobin levels can be increased in inflammatory conditions, of the two patients in our study who were admitted with acute respiratory failure neither was noted to have abnormally elevated baseline serum haptoglobin levels.

For the twenty-one patients, serum haptoglobin levels taken at pretransfusion, immediately after, 24 hours and forty eight hours after transfusion were examined for significant differences using analysis of variance with repeated measures. No significant differences were found after adjusting using Greenhouse-Geisser's estimate, DF = 3.60, *F* = 1.26, *P* = .28. 

**Figure 1:**
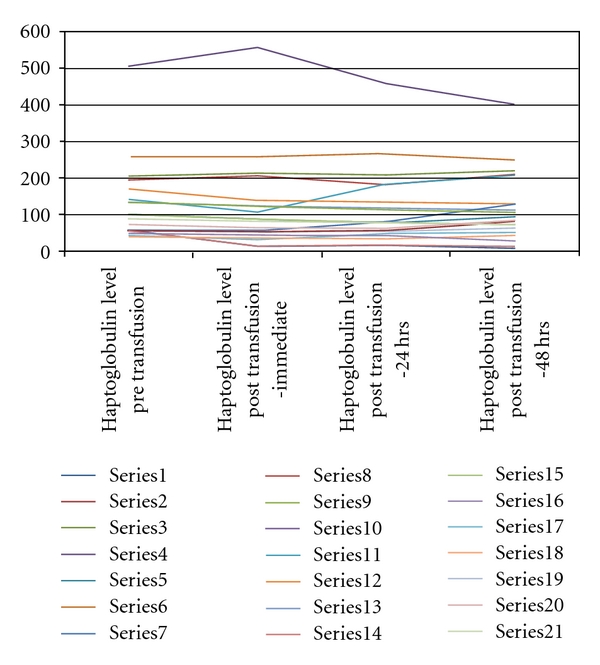
Haptoglobin levels for the 21 patients at various time intervals.

Figure 1 and Table 1 demonstrate the haptoglobin levels for all twenty-one patients at the various time intervals.  Table 2 depicts the mean, standard deviation and mean differences in haptoglobin levels at each time interval. The differences were examined using univariate paired *t*-test with only a significant difference found from pretransfusion haptoglobin levels to the haptoglobin levels immediately after transfusion (*P* = .041, two sided).

In the twenty-one patients haptoglobin levels before transfusion were all within the normal range. Immediately after transfusion three patients had levels below normal and two of these patients had a persistent decline in haptoglobin levels at 24 and 48 hours. In addition, one patient had a decline in the serum haptoglobin level at 24 hours and one at 48 hours. Both these patients had normal levels at other time periods. 

We also noted that a total of four patients received blood greater than 30 days old. Of these 2 had haptoglobin levels less than 36 mg/dL at 48 hours after transfusion. This was not statistically significant at *P* = .07, Fisher Exact. 

We also observed that three patients with a low normal haptoglobin at baseline (less that 60 mg/dL) had a level less than 36 mg/dL at 48 hours. No patient with a level above 60 mg/dL before transfusion had an abnormal level of less than 36 mg/dL at 48 hours. This was significant at *P* = .02, Fisher Exact. 

Our study was not powered to look at the effect of massive amounts of PRBC transfusion on serum haptoglobin levels. However, we did note that of the three patients who received more than two units of PRBC, only one had a posttransfusion level that was below normal and this drop was seen at 48 hours after transfusion. This patient had received four units of PRBC all of which were more than 30 days old.

## 5. Discussion

Measurement of the serum haptoglobin is used to diagnose hemolysis. However, the accuracy of measuring serum haptoglobin in patients who have received PRBC is not known. 

In our study we note a statistically significant drop in the mean serum haptoglobin levels immediately after transfusion (*P* = .041). However this drop except when the pretransfusion haptoglobin levels are below 60 mg/dL had no clinical relevance since the absolute numbers continued in the normal range. In their study, Nishiyama and Hanaoka reported an inverse proportionality between serum haptoglobin levels and the age of transfused blood [8]. They observed that the serum haptoglobin levels decreased with transfusion of at least 1000 ml or more of whole blood with a mean storage time of 12.2 days. In our study the maximum amount of transfused PRBCs was 800 ml (two patients). None of our study patients received whole blood. However, for indications such as anemia, transfusion of whole blood as well as transfusion of massive amount of PRBCs is rare.

Our study was not powered to study the effect of massive amounts of PRBC transfusion on serum haptoglobin levels. However, in the three patients who received more than two units of PRBCs we noted a drop in serum haptoglobin in only one patient who had received four units of blood, all of which were more than thirty days old. This drop was noted at 48 hours. The half-life of the haptoglobin-hemoglobin complex is a few minutes, so this drop in the serum haptoglobin at 48 hours cannot be explained by the rapid clearing of the hemoglobin-haptoglobin complex formed after transfusion. 

We did observe a small decline in serum haptoglobin levels to below normal in two of the four patients who were transfused with blood more that 30 days old. These two patients were also noted to have a low normal pretransfusion serum haptoglobin level. The cause for this decline remains unclear. However this decline was transient and did not appear to significantly undermine the utility of serum haptoglobin measurement in the diagnosis of hemolysis in PRBC recipients.

## 6. Conclusion

These results suggest that serum haptoglobin levels can be used as a tool to assess hemolysis in patients who have received multiple units of PRBCs. A small, transient decline in serum haptoglobin levels can be noted in these patients especially if the pretransfusion haptoglobin levels are low normally. Although the cause for this is unknown, this may suggest a low-grade hemolysis and this decline, unless persistent, may not be clinically relevant. 

Also, there has been considerable interest in the effects of free hemoglobin on nitric oxide and the resulting vascular complications seen in both acute and chronic hemolysis [9, 10] and these findings merit further investigation.

This was a small prospective study aimed at identifying the role of serum haptoglobin measurement in patients receiving multiple units of blood transfusion. Further studies, with a larger sample size, aimed at studying the serum-free hemoglobin levels and serum haptoglobin levels are needed to corroborate our findings and to further evaluate if the amount of transfusion or age of transfused blood has any effect on the serum haptoglobin levels.

##  Conflict of Interests

The authors have no conflicts of interest to disclose.

## Figures and Tables

**Table 1:** Haptoglobin levels for all the patients.

S. no.	Haptoglobin level pretransfusion	Haptoglobin level after transfusion—immediate	Haptoglobin level after transfusion—24 hrs	Haptoglobin level after transfusion—48 hrs
1	58.3	15.2	18.3	10.5
2	57.3	55.8	57.6	83.3
3	207	215	211	221
4	505	556	460	403
5	102	90	77.5	96.3
6	259	258	268	250
7	59	57.7	82	130
8	198	206	182	210
9	135	124	114	108
10	173	140	134	131
11	142	108	185	208
12	173	140	135	131
13	135	124	114	114
14	58	15	18	15
15	102	90	77	65
16	50	45	45	30
17	44	33	50	53
18	40	38	35	45
19	60	55	55	65
20	75	65	65	85
21	90	85	80	75

**Table 2:** Mean, standard deviation (SD) and mean differences in haptoglobin levels at each time interval. The differences were examined using univariate paired *t*-test with only a significant difference found from pre haptoglobin levels and the haptoglobin levels immediately after transfusion significant at *P* = .041 (two sided).

	MEAN	SD	95% LCL	95% UCL	Number of patients with <36	Mean differences	*P* values
Pre Level	129.64	106.14	81.32	177.96	0		
One Hour	119.79	119.73	65.29	174.29	3	9.85	.041
24 hours	117.30	102.59	70.60	164.00	2	2.49	ns
48 hours	120.43	93.80	77.7	163.13	3	3.1	ns
